# Editorial: Emotionally-centred perinatal care, practices and experiences

**DOI:** 10.3389/fgwh.2026.1839657

**Published:** 2026-04-21

**Authors:** Orli Dahan, Gill Thomson, Claire Feeley

**Affiliations:** 1 Tel-Hai College, Upper Galilee, Israel; 2 University of Lancashire, Preston, United Kingdom; 3 King’s College London, London, United Kingdom

**Keywords:** emotion, maternity, maternity care, perinatal, positive care

Childbirth is a biopsychosocial and physiological event; however, it is often framed within a clinical or medical lens with a hyperfocus on physical processes. While it may require coordination, surveillance, and intervention (1), the experience of birth far exceeds this clinical lens. For women and birthing people, childbirth is often experienced as a profound emotional and relational event: one that engages vulnerability and strength, fear and surrender, intimacy and interdependence (2). To focus solely on the physical processes may mask what makes childbirth life affirming, tolerable, or traumatic (3).

For decades, the dominant bio-medical model in obstetrics has focused almost exclusively on morbidity and mortality, long organised around the pervasive mantra that “a healthy mother and a healthy baby” are the paramount outcomes. A framing that reflects what Barbara Katz Rothman (4) describes as the expansion of a “biomedical empire” – a system in which biomedicine functions not only as a clinical enterprise but as an economic, moral, and cultural authority, often reducing complex human experiences to biological metrics and risk management.

This biomedical logic can marginalise women's embodied, relational, and experiential knowledge. However, as contemporary research and voices from the field increasingly demonstrate, physical survival alone is insufficient as a marker of the quality of care. A positive birth experience has been described as

“a woman's experience of interactions and events directly related to childbirth that made her feel supported, in control, safe, and respected; a positive childbirth can make women feel joy, confident, and/or accomplished and may have short and/or long-term positive impacts on a woman's psychosocial well-being. Leinweber et al. (5)”

A positive birth is not a luxury; it is a critical springboard for the transition to parenthood, positive maternal mental health and family wellbeing (6, 7). With global calls to improve maternal-neonatal outcomes, and to move beyond “survival” towards “thriving” (8), an emotional safety lens becomes paramount to this endeavor (9).

This Research Topic, *Emotionally-centred Perinatal Care, Practices and Experiences*, consolidates and extends this shift through 16 contributions spanning original empirical studies, theoretical analyses, and perspective papers. Taken together, the collection proposes that childbirth can be understood as an *emotional ecology*: a complex interplay of embodied and neurobiological processes, relational and communicative practices, sensory and environmental cues, and organisational structures that shape safety, agency, wellbeing, and meaning in childbirth.

## Psychological wellbeing and positive experiences as outcomes in their own right

One of the key themes from the collection relates to how emotionally-centred care is protective across the perinatal period. Tabib et al.'s longitudinal study of antenatal relaxation education demonstrates that even a single session can significantly enhance childbirth self-efficacy, mental wellbeing, and reduce fear and anxiety, with effects extending into the early postpartum period. Complementing this empirical evidence, Karlsdottir and Leap argue for the recognition and routine measurement of women's childbirth experiences as core quality indicators in maternity care, emphasising that positive birth experiences support empowerment, bonding, and long-term mental health, while negative experiences can contribute to diminished self-esteem and trauma-related outcomes.

## From trauma-informed safety to respectful, non-violent care

Another central strand in the collection foregrounds trauma-informed maternity care and respectful practice, emphasising emotional safety as foundational to childbirth. Montgomery and Duckworth highlight how a “failure to listen” is especially consequential for survivors of child sexual abuse, while Cull et al. introduce the EMPATHY framework for routine trauma discussions designed to reduce re-traumatisation. These approaches intersect with explicit engagement with obstetric violence; Reyes-Amargant et al. reveal the epistemic and moral tensions, such as power asymmetries and lack of consent, that sustain non-respectful care. Critically, these contributions argue that preventing obstetric violence and avoiding re-traumatisation are essential components of clinical and emotional safety. As such, “woman-centered care” needs to be operationalised through practices that promote autonomy and agency, helping to mitigate distress, and optimise a care environment with the potential for healing and restoration, particularly for those with histories of trauma. Importantly, this Topic pushes beyond an individualised view of compassion; Redhead's analysis proposes therapeutic jurisprudence as a lens to address the organisational structures that often undermine compassionate care. Together, these works reframe emotionally-centered care as both an ethical necessity and a system-level commitment.

## The sensory and environmental field of birth

A third cluster in this collection explores how sensory and environmental conditions modulate emotional states, coping, and labour processes. Rather than seeing the birth environment as a neutral clinical backdrop, contributions here reveal how textures, lighting, and spatial boundaries actively mediate the emotional landscape. For instance, Dahan and Goldberg's comparative study shows that when compared to women who gave birth in hospitals, women who birthed at home reported significantly higher “flow” states- a psychological state where a birthing person is fully immersed, focused, and absorbed. Complementing this, Albo et al. report that dim lighting in hospital settings is associated with higher rates of vaginal birth and fewer perineal tears, pointing to light as a modifiable feature that has both physiological and experiential relevance.

The contributions further illustrate how the environment can be actively reconfigured even within high-surveillance spaces. Clossick's self-analysis of a high-risk labour ward demonstrates how a low-tech intervention – creating a “sanctuary” behind a cloth screen – can support privacy and autonomy, suggesting that micro-spatial changes may generate meaningful cultural shifts. Other papers highlight more sensory-oriented rather than environmental factors; Haslund-Thomsen et al. describe how auricular acupuncture reduces stress for parents in the NICU, while Crowther et al. explore Havening as a psycho-sensory therapy for emotional resilience. Overall, these works point to sensory and environmental features through which safety and physiological processes are either supported or disrupted.

## Relational care, collective learning, and childbirth agency

A fourth strand concentrates on the relational fabric of care and the social mechanisms through which agency is enabled. Across diverse settings – labour wards, antenatal groups, and institutional reform efforts – the contributions converge on a shared insight: agency in childbirth is not an individual state but a relational achievement. Skeide's ethnographic work in German midwifery settings and De Quattro's study of antenatal preparation both demonstrate how agency emerges through interactional practices rather than through isolated decision-making. While Skeide analyses how midwifery techniques such as “spooning” and positioning foster “care attachments” that shape bodies-in-labour, De Quattro shows how storytelling, group-led knowledge practices, and even “care-full absences” allow birthing people to interpret and inhabit childbirth as meaningful. In both cases, agency is enacted through attunement, embodied responsiveness, and shared sense-making. This relational dynamic is further extended in McCourt et al.'s evaluation of group antenatal care (“Pregnancy Circles”), where empowerment arises from participatory learning, continuity, and mutual recognition among women and professionals.

Importantly, relational agency also includes staff experience. Brimdyr et al.'s examination of skin-to-skin implementation through Antonovsky's “sense of coherence” framework shows how staff comprehensibility, manageability, and meaningfulness are preconditions for sustaining parent and infant relational care. Redhead's socio-legal analysis also highlights how rigid hierarchies and cultures of blame constrain the emotional capacities of caregivers. Together, these studies highlight how emotional or relational care cannot be sustainably implemented within emotionally depleted systems.

## Conceptual horizons: birth as interdependent, embodied, and meaning-bearing

Finally, beyond empirical findings, the Topic includes work that expands the conceptual vocabulary available for thinking about emotionally-centred care. Sadler and Cohen Shabot draw on the “fungal turn”, using mycelial networks as a metaphorical and conceptual resource for reframing childbirth as relational, interdependent, and permeable. These insights challenge individualistic models of agency, highlight how trust and safety can enable surrender rather than hypervigilant control. This conceptual reframing challenges dominant liberal and biomedical imaginaries of birth as an individualised event, as well as the epistemological assumptions that privilege control, separability, and risk management, instead positioning childbirth as an emergent relational field in which agency is co-constituted across bodies, environments, and care relations.

## Toward an agenda for emotionally-centred perinatal care

Collectively, the 16 contributions in this Research Topic span empirical, theoretical, and experiential contributions across multiple disciplines. They suggest that emotionally-centred perinatal care is a paradigmatic reorientation with implications for research, practice, training, and policy. Seen through a relational ontology, insights from this collection position socio-spatial sanctuaries, sensory ecologies, midwifery care attachments, and group-based knowledge practices not as isolated interventions, but as expressions of an interdependent birth ecology. This collection offers an invitation: to take the emotional dimensions of childbirth seriously – as a core determinant of safety, agency, and wellbeing. Emotionally-centred perinatal care reframes birth as an embodied and relational process shaped by sensory environments, trauma histories, caregiving practices, collective learning, and organisational structures. In doing so, the Topic offers both a consolidated knowledge base and a forward-looking agenda for building more humane, responsive, and respectful perinatal care.
